# Influences on androgen deprivation therapy prescribing before surgery in high‐risk prostate cancer

**DOI:** 10.1002/bco2.411

**Published:** 2024-07-14

**Authors:** Jennifer Dunsmore, Eilidh Duncan, Sara J. MacLennan, James N'Dow, Philip Cornford, Francesco Esperto, Nicola Pavan, María J. Ribal, Monique J. Roobol, Ted A. Skolarus, Steven MacLennan

**Affiliations:** ^1^ Academic Urology Unit University of Aberdeen Aberdeen UK; ^2^ Health Service Research Unit University of Aberdeen Aberdeen UK; ^3^ Liverpool University Hospitals Liverpool UK; ^4^ Department of Urology Campus Biomedico University of Rome Rome Italy; ^5^ Urology Section, Department of Surgical, Oncological and Stomatological Sciences University of Palermo Palermo Italy; ^6^ Uro‐Oncology Unit, Hospital Clinic University of Barcelona Barcelona Spain; ^7^ Department of Surgery, Urology Section Erasmus University Medical Center Rotterdam, Cancer Institute Rotterdam The Netherlands; ^8^ VA Ann Arbor Healthcare System Ann Arbor Michigan USA; ^9^ The University of Chicago Chicago Illinois USA

**Keywords:** behaviour change, de‐implementation, determinants, influences, intervention, neoadjuvant androgen deprivation therapy, practice change, qualitative methods, strategies

## Abstract

**Objectives:**

To understand how best to further reduce the inappropriate use of pre‐surgical androgen deprivation therapy (ADT), we investigated the determinants (influences) of ADT prescribing in urologists in two European countries using an established behavioural science approach. Additionally, we sought to understand how resource limitations caused by COVID‐19 influenced this practice. Identification of key determinants, of undistributed and disrupted practice, will aid development of future strategies to reduce inappropriate ADT prescribing in current and future resource‐limited settings.

**Participants and Methods:**

We conducted semi‐structured qualitative interviews with urologists practicing in Italy and the UK from February to July 2022. Interviews focussed on undisrupted (usual) practice and disrupted practice (changes made during COVID‐19 restrictions). Codes were generated inductively and were mapped to the 14 domains of the Theoretical Domains Framework. Relevant domains of influence were identified, and the similarities and differences between the UK and Italy were distinguished.

**Results:**

We identified 10 domains that were influential to ADT prescribing in the UK and eight in Italy. The role of guidance and evidence, the cancer care setting, the patients and the urologist's beliefs and experiences were identified as areas that were influential to ADT prescribing before surgery. Twenty‐one similarities and 22 differences between the UK and Italy, for usual and COVID‐19 practice, were identified across these 10 domains.

**Conclusion:**

Similarities and differences influencing ADT prescribing prior to surgery should be considered in behavioural strategy development and tailoring to reduce inappropriate ADT use. We gained an understanding of usual, undistributed care and resource‐limited or disrupted care due to COVID‐19 in two European countries. This gives an indication of how influences on ADT prescribing may change in future resource‐limited circumstances and where efforts can be focused now and in future.

## 1 INTRODUCTION

The impact of COVID‐19 in Europe caused many countries to restrict or alter their cancer services^1^ with a significant impact on surgical capacity.^2^, ^3^ Alternate practices, such as prescribing neoadjuvant androgen deprivation therapy (ADT) in prostate cancer patients, were explored in place of unavailable surgery.^4^ Unnecessary ADT use in Europe had already been highlighted as an issue prior to COVID‐19 and was prioritised for de‐implementation.^5^ (Re)establishment of evidence‐based oncology practices can be accommodated with the development of theory‐informed de‐implementation strategies, to ensure current evidence‐based guidelines are followed, resources are used efficiently and any unnecessary harm to patients is reduced.

The European Association of Urology's (EAU's) IMpact Assessment of Guidelines Implementation and Education (IMAGINE) group^6^ performed an audit across Europe and found non‐adherence to pre‐surgical ADT guidelines ranged 0% to 32% across risk groups. The most variability was identified in patients with high‐risk prostate cancer, where non‐adherence ranged 0% to 43%.^7^ The variability in ADT practice, especially in patients with high‐risk prostate cancer, had been previously highlighted across multiple European countries before this audit.^5^, ^8^, ^9^ The common reasons reported in the audit for offering ADT before surgery included the healthcare professional's belief that ADT could improve cancer outcomes, reduce the volume of the tumour or reduce the chance of positive margins in surgery. Additionally, patients changing their treatment preference (i.e., high‐risk patients switching from receiving appropriate ADT before radiotherapy to the surgery option) was also frequently reported.^7^

However, since this audit, the pandemic severely impacted the availability of surgical resources for cancer treatment, initially in Italy^10^ and then the rest of Europe.^11^ These disruptions prompted (inter)national urological societies to review their advice.^12^ The strong recommendation to not offer neoadjuvant ADT before surgery was maintained by the EAU.^13^ In the UK, the British Association of Urological Surgeons (BAUS) offered additional recommendations which differed from EAU's guidance. Recommendations stated that high‐risk and unfavourable intermediate non‐metastatic prostate cancer patients “should be offered on hormone therapy [ADT] … until a time is available to offer them curative therapy (radical prostatectomy/radical radiotherapy)”.^4^ Other urological professional groups also suggested starting ADT with a view to offer curative treatment options when available.^10^, ^14^ In contrast, other societies, such as the Italian Society of Urology (SIU), remained consistent with the EAU's strong recommendation against the use of neoadjuvant ADT before surgery.^15^

A clear understanding of the influences on ADT prescribing is essential to compose strategies to eradicate this unnecessary practice.^16^ Although practice during COVID‐19 was not an original focus of the initial study plan, the disruptions to prostate cancer care provided a further dimension to understand urologist prescribing behaviours and any potential legacies of changes to practice from COVID‐19.^17^ This study uses a behavioural science approach to explore the influences that underpin a urologist's decision to prescribe ADT in two European countries—the UK, where the national society suggested the use of ADT during COVID‐19, and Italy, where the national society's recommendations aligned with the EAU. As much of the variation in ADT practice is around the treatment of patients diagnosed with high‐risk prostate cancer, the focus of this study was to understand the influences on urologists' recommendation or prescription of neoadjuvant ADT in patients diagnosed with high‐risk prostate cancer opting for or scheduled for surgery.

## 2 METHODS

### 2.1 Design

A cross‐sectional descriptive qualitative interview study was conducted in the UK and Italy. This study is reported according to the COREQ statement checklist (Data S1).

### 2.2 Participant identification

Urologists involved or responsible for treatment decisions for patients with high‐risk localised prostate cancer, during and following the easing of COVID‐19 restrictions, were recruited. This eligibility criteria were verified by email before the interview. UK‐ and Italian‐based registrars and consultants (or equivalents) were recruited through professional networks. Recruiting via social media was also used. Those interested emailed the researcher.

National COVID‐19 lockdowns began in early 2020 in the UK^18^ and Italy.^19^ We define the start of COVID‐19 as March 2020 for both countries. Presently, COVID‐19 does not have a clear ‘end date’; however, restrictions across Europe reduced or ended in 2022, and health care became less restricted.

### 2.3 Data collection

One‐to‐one semi‐structured interviews were completed over the phone or MS Teams, by an experienced qualitative researcher with an MSc in Health Psychology (J.D., PhD student, female). Interviews were conducted in English. All participants were sent a participant information sheet and completed a consent form before the interview. Interviews were audio‐recorded, transcribed verbatim and analysed using QSR Nvivo. Interviews ranged 14–52 min. Interviews were conducted from February to July 2022.

This study was approved by the University of Aberdeen Life Sciences and Medicine Ethics Review Board (SERB) reference: SERB/2021/11/2217.

### 2.4 Materials

An interview schedule (Data S1) was developed based on the Theoretical Domains Framework (TDF).^20^ The TDF synthesises 33 behavioural theories into 14 domains of influence. These domains explore potential barriers and facilitators experienced by healthcare professionals regarding a specified target behaviour, such as prescribing pre‐surgery ADT to patients with high‐risk non‐metastatic prostate cancer. The 14 domains and definitions are listed in Table S1.

The interview schedule took a pragmatic approach as opposed to a traditional TDF interview of asking multiple question for each domain in turn. We covered all TDF domains by asking more ‘general’ questions—for example: “was there anything COVID brought about that affected [ADT prescribing]?”, followed by further questions or prompts to understand areas of influence. We opted for this approach to accommodate the participants as we were aware their time was limited. Questions about treatments during the impact of any COVID‐19 restrictions (starting from March 2020) and following the easing of restrictions (which differed at each hospital) were included. An interview schedule from a similar study conducted in the United States was considered to ensure potentially relevant themes were also explored in the European setting.^21^ Again, due to urologist's availability at the time of piloting, the interview schedule was piloted with two urologists, one UK and one US‐based.

### 2.5 Analysis

An inductive content analysis approach was used. Coding decision rules were updated iteratively. Initial coding was completed by one researcher (J.D.) and checked independently by two researchers (S.M. and E.D.). Beliefs that were identified in the transcripts were grouped and mapped to the TDF domains.

Domains were assessed for relevance at country level, which meant that domains relevant to the UK may not have been relevant to Italy. Each of the domains and the associated beliefs were analysed for relevance using an establish criteria.^22^ Domains were considered relevant if their associate beliefs were of high frequency (≥70% participants), had conflicting belief statements and/or had strong beliefs that could impact behaviour.

Overarching narrative titles were used to contextualise the results. Main similarities and differences were identified between the countries. There is not a standard method to identify similarities and differences in influences on a behaviour therefor the research team devised a criterion. A similarity was identified where the percentage of participants referring to a belief within relevant domains were less than 20% discrepant (e.g., 90% and 100%, has a discrepancy of 10%). Whereas a difference was identified where the percentage of participants referring to a belief was more than 20% discrepant, (e.g., 90% and 10%, has a discrepancy of 80%). We considered our sample size and agreed that a 20% threshold would reasonably indicate these features. Additionally, similarities and differences could be acknowledged based on the assessment of the content of the data within the codes, despite the percentage difference.

### 2.6 Data saturation

The sampling followed the 10 + 3 rule.^23^ In the UK sample, following Interview 10, no new beliefs were identified, and the last identified belief was coded in Interview seven. The last identified belief in the Italian sample was coded in the seventh interview (numbered 19). Due to time constraints, 10 interviews were conducted in Italy.

## 3 RESULTS

### 3.1 Characteristics of participants

Twenty‐two participants were interviewed, 12 participants practiced in the UK and 10 practiced in Italy. Eleven consultants and one registrar were based in the UK, with an average of 7 years of practice (which ranged 1–15 years). Seven Consultants, and three Registrars (or equivalent) were based in Italy, with an average of 4 years of practice (which ranged 1–13 years).

### 3.2 Characteristics of hospital sites

The UK‐based participants were from nine sites located in England, Scotland and Wales. The Italian‐based participants were from eight sites, which spanned north, central and south Italy. Sites were generally reported as academic and high volume. See Table 1 for a summary of characteristics of sites in each country.

**TABLE 1:** Site characteristics and impact to surgery due to COVID‐19 in the UK and Italy.

Participant number	Location	Type of Hospital	Volume	Length of delay to surgery	Hormones considered
UK					
1	Scotland	Academic	High	2 months	Yes
2	Scotland	Academic	High	1.5 months	Yes
3	England	Academic	High	3–5 months	Yes
4	England	Academic	High	No delay	No
5	England	Academic	High	2–3 months	Yes
6	England	Academic	High	<1 month	Yes
7	Wales	Academic	High	No delay	No
8	England	Academic	High	3 months	Yes
9	England	Academic	High	6 months (2020), 2 months (2021)	Yes
10	England	Academic	High	No delay	No
11	Scotland	Academic	High	6 months	Yes
12	England	Academic	High	No delay	No
Italy					
13	North	Academic	High	No delay	No
14	South	Academic	High	3–6 months	No
15	Central	Academic	High	No delay	No
16	North	Academic	High	4 months	Yes
17	North	Academic	High	Unclear	Yes
18	South	Mixed	High	2 months	No
19	North	Academic	High	4 months	Yes
20	Central	Academic	High	3–4 months	Yes
21	Central	Academic	High	No delay	No
22	North	Private	Medium	No delay	No

During COVID‐19, eight UK participants reported delays to surgery (ranging 1–6 months) and considered neoadjuvant ADT while surgery was delayed. In Italy, five participants reported delays (ranging 2–6 months), and four considered ADT in this time.

### 3.3 Relevant domains for the UK

Ten domains, consisting of 65 belief statements, were considered relevant to ADT prescription behaviour in the UK. Relevant domains were (1) Behavioural Regulation, (2) Beliefs about Consequences, (3) Emotion, (4) Environmental Context and Resources, (5) Goals, (6) Intention, (7) Knowledge, (8) Memory, Attention and Decision Processes, (9) Social Influences and (10) Social/Professional Role and Identity. Of the 65 belief statements identified, 33 related to usual, non‐disrupted practice. Thirty‐two belief statements (of 65) related to practice during COVID‐19 restrictions.

### 3.4 Relevant domains for Italy

Eight domains, consisting of 55 belief statements, were considered relevant to ADT prescription behaviour in Italy. Domains were (1) Behavioural Regulation, (2) Environmental Context and Resources, (3) Goals, (4) Intention, (5) Knowledge, (6) Memory, Attention and Decision Processes, (7) Social Influences and (8) Social/Professional Role and Identity. Of the 55 belief statements identified, 31 related to usual, non‐disrupted practice. Twenty‐four belief statements (of 55) related to practice during any COVID‐19 restrictions. See Table 2 for comparison of the relevant domains for both countries and periods of practice.

**TABLE 2:** Domains identified relevant to neoadjuvant ADT prescribing before surgery for Usual and COVID‐19 practice in the UK and Italy.

Domains	UK		Italy	
	Usual practice	COVID‐19 practice	Usual practice	COVID‐19 practice
Behavioural Regulation	✓	✓	✓	✓
Environmental Context and Resources	✓	✓	✓	✓
Intention	✓	✓	✓	✓
Knowledge	✓	✓	✓	✓
Memory, Attention and Decision Processes	✓	✓	✓	✓
Social/Professional Role and Identity	✓	✓	✓	✓
Social Influences	✓	✓	✓	✓
Belief About Consequences	✓	✓	✕	✕
Goals	✕	✓	✕	✓
Emotion	✕	✓	✕	✕
Beliefs About Capabilities	✕	✕	✕	✕
Optimism	✕	✕	✕	✕
Reinforcement	✕	✕	✕	✕
Skills	✕	✕	✕	✕

### 3.5 Similarities and differences in COVID‐19 practice

#### 3.5.1 Similarities

During COVID‐19, in both the UK and Italy, participants were generally aware of any changes to management and prioritisation guidance, where relevant. There was a consensus across both countries that any changes in usual management guidelines should only be used where COVID‐19 impacted usual treatments. There were also similarities in the beliefs related to the cancer care setting. Participants reported that resources such as available staff, theatres and bed space to offer treatment options were impacted by COVID‐19, although this was to varying degrees within and between countries, from a complete suspension to alteration of non‐urgent services that remained available. One UK‐based participant experienced long surgical wait list prior to the pandemic due to lack of available resources and provided pre‐surgical ADT to manage patients but acknowledged this was not guideline adherent. Four participants, in both the UK and Italy, reported that private hospitals or resources were used to help manage prostate cancer patients. It was recognised in both countries that resources used for surgery were allocated to COVID‐19 efforts; however, participants felt that oncology generally remained a priority.

#### 3.5.2 Differences

There was a noticeable difference between the UK and Italy in the knowledge of the changes to guidance regarding ADT use. In the UK, it was well recognised that BAUS offered a recommendation to consider ADT prior to curative treatment. However, Italian‐based urologists were less likely to be aware of any changes to guidance as their national society maintained the usual recommendation. Patients in the UK were not offered the usual options for treatment and urologists thought that pre‐surgical hormones were considered a reasonable ‘holding measure’, this was not the case in Italy. Only a few urologists based in Italy stated they ‘may consider’ ADT as opposed to actively using it. The majority of differences identified related to the urologists' personal beliefs and experience of ADT. In the UK, prescribing ADT during the COVID‐19 was influenced by the belief in the outcomes that may be achieved with pre‐surgical ADT, including ‘buying’ time and ‘controlling’ the cancer. UK‐based urologists' decisions were also uniquely influenced by emotions, some urologists reported feeling stress and worry around the change in these treatment options, particularly at the beginning of the pandemic. The consideration of outcomes and impact of emotions were not considered influential to Italian‐based urologists' decisions, as they were not frequently mentioned or deemed unlikely to impact prescribing behaviour during COVID‐19. See Table 3 for the main similarities and differences for COVID‐19 practice. Interview quotations can be found in Table S2.

**TABLE 3:** Main similarities and differences of beliefs across the UK and Italy for COVID‐19 practice.

	UK, *n* (%)	Italy, *n* (%)
**Similarities**		
The role of guidelines and evidence		
COVID‐19; I used guidance or adapted COVID‐19 guidance to guide my practice (Knowledge)	11 (92)	10 (100)
COVID‐19; We will only offer hormones or follow COVID‐specific guidance if or when needed (Intention)	10 (83)	7 (70)
COVID‐19; We prioritised patients according to available prioritisation guidance for surgical patients (Environmental Context and Resources)	9 (75)	7 (70)
The cancer care setting and patients		
COVID‐19; I had to consider the surgical capacity to offer surgery (Environmental Context and Resources)	10 (83)	10 (100)
COVID‐19; Patients, for the most part, were understanding of the changes made to treatment options (Social Influences)	6 (50)	5 (50)
COVID‐19; Asides from the pandemic, resources were allocated to cancer care (Environmental Context and Resources)	5 (42)	6 (60)
COVID‐19; Cancer targets relating to treating prostate cancer were or were not ‘dropped’ during COVID‐19 (Behavioural Regulation)	3 (25)	2 (20)
COVID‐19; We used private hospitals to offer surgery to mitigate any delays (Environmental Context and Resources)	2 (17)	2 (20)
**Differences**		
The role of guidelines and evidence		
COVID‐19; Guidelines specified use of hormones (ADT) before surgery (Knowledge)	11 (92)	1 (10)
COVID‐19; I referred to available guidance to help mitigate any delays due to COVID‐19 (Environmental Context and Resources)	10 (83)	6 (60)
COVID‐19; Anaesthetic guidelines delayed surgery to ensure 7 weeks of recovery from COVID‐19 (Environmental Context and Resources)	4 (33)	0 (0)
The cancer care setting and patients		
COVID‐19; Eligible patients were not given equal option between surgery or radiotherapy treatments (Environmental Context and Resources)	9 (75)	4 (40)
The urologist's beliefs and experience		
COVID‐19; I would offer hormones (ADT) where delays in surgery were experienced or anticipated (Environmental Context and Resources)	Yes: 8 (67) No: 2 (17)	Yes: 4 (40) No: ‐ (‐)
COVID‐19; Other cancers or illnesses were prioritised over localised prostate can (Goals)	9 (75)	5 (50)
COVID‐19; I think that other urologists in my country practiced the way I did in COVID‐19 (Social Influences)	9 (75)	3 (30)
COVID‐19; ADT could prevent the cancer from getting worse while surgery was delayed (Belief About Consequences)	8(67)	‐ (‐)
COVID‐19; Giving ADT before surgery allows you to buy time (Belief About Consequences)	5 (42)	‐ (‐)
COVID‐19; There remained an urgency to treat high‐risk prostate cancer (Goals)	6 (50)	8 (80)
COVID‐19; I found treatment decisions during COVID‐19 stressful (Emotion)	4 (33)	‐ (‐)
COVID‐19; I felt it was risky to make patients wait without treatment (Emotion)	3 (25)	‐ (‐)
COVID‐19; I worry that offering hormones (ADT) caused harm (Emotion)	3 (25)	‐ (‐)

### 3.6 Similarities and differences in usual practice

#### 3.6.1 Similarities

In the UK and Italy, all participants reported their practice was informed by usual (inter)national practice guidelines, as they felt guidance offered acceptable courses of action. The majority of participants do not intend to recommend or prescribe pre‐surgical ADT. Participants reported that this practice was not routine for them; however, there were a couple of participants in both countries that stated they had seen pre‐surgical ADT being used previously but this has decreased over time.

Participants felt other urologists in their country follow (inter)national practice guidelines also. It was also acknowledged in both countries that adhering to guidance is not mandatory, and it is acceptable to deviate where required and make treatment recommendations based on clinical judgement. There was an understanding that other urologists may justify their ADT practice with, for example, local cohort data, patient preferences or ‘selected’ cases. Again, urologists based in both countries had mixed views if justifications such as these were acceptable.

Although only mentioned a handful of times, participants in both countries referred to the legitimacy of the evidence underpinning the current guidance, reporting that studies were out of date or not well conducted. Participants in both countries report that in usual, non‐disrupted practice, there are no alternative therapies that could be offered in the neoadjuvant setting. The only widely accepted reason for offering pre‐surgical ADT was during a clinical trial.

The influence of the patient's preferences and concerns heavily influenced treatment recommendations in both countries. If patients were unsure, needed time to decide or were extremely anxious, ADT may be offered even if the patient opts for surgical treatment or is yet to decide on a curative treatment. Participants in both countries appreciated that patients have the ‘ultimate’ decision and are allowed to change their mind mid‐treatment.

#### 3.6.2 Differences

Participants based in Italy believed that an academic environment facilitates guideline adherence, this was not mentioned in the UK. Academic environments have staff members involved with research and/or guidelines, and has established processes, such as MDT meetings, to ensure guidelines are considered. Italian‐based participants thought MDTs were essential to ratify or aid decision‐making. In the UK, MDTs were seen as part of the process and participants referred more often to the evidence that underpinned the guidelines as justification of their practice. More Italian‐based participants, felt that their practice was in line with their departmental colleagues, as compared to their UK counterparts.

The beliefs about the consequences of pre‐surgical ADT were only deemed relevant to UK‐based participants as there was less reference to the positive or negative effects of pre‐surgical ADT from Italian‐based participants. The beliefs that ADT has side effects and may impact future treatment options were frequently mentioned, by UK‐based participants as considerations when prescribing ADT, however there were inconsistent views if ADT had an impact on the surgery. See Table 4 for the main similarities and differences for usual practice. Interview quotations can be found in Table S3.

The beliefs about the consequences of pre‐surgical ADT were only deemed relevant to UK‐based participants as there was less reference to the positive or negative effects of pre‐surgical ADT from Italian‐based participants. The beliefs that ADT has side effects and may impact future treatment options were frequently mentioned, by UK‐based participants as considerations when prescribing ADT, however there were inconsistent views if ADT had an impact on the surgery. See Table 4 for the main similarities and differences for usual practice. Interview quotations can be found in Table S3.

**TABLE 4:** Main similarities and differences of beliefs across the UK and Italy for usual practice.

	UK, *n* (%)	Italy, *n* (%)
**Similarities**		
The role of guidelines and evidence		
I believe the guidelines are followed in my country (Social influence)	12 (100)	10 (100)
My ADT practice is informed by guidelines (Behavioural Regulation)	12 (100)	10 (100)
There are no alternatives to ADT before surgery (Knowledge)	12 (100)	7 (70)
I find usual guidance is acceptable (Social/Professional Role and Identity)	10 (83)	10 (100)
I am allowed to deviate from the guidance if required (Social/Professional Role and Identity)	6 (50)	5 (50)
Updated research should be conducted in the neoadjuvant setting (Knowledge)	4 (33)	3 (30)
The cancer care setting and patients		
Patients have the ultimate decision/heavily influence on their treatment (Social Influence)	11 (92)	9 (90)
Treatment options for every cancer patient are discussed in an MDT meeting (Environmental Context and Resources)	10 (83)	10 (100)
ADT before surgery could be considered in a clinical trial (Environmental Context and Resources)	4 (33)	4 (40)
The urologist's beliefs and experience		
I do not offer ADT before surgery in my usual practice (Intention)	10 (83)	7 (70)
Other urologists may or may not have justifiable reasons to use ADT before surgery (Social Influences)	Justifiable: 6 (50) Not justifiable: 3 (25)	Justifiable: 5 (50) Not justifiable: 4 (40)
ADT before surgery has not been a routine treatment in the time or places I have practiced (Environmental Context and Resources)	9 (75)	8 (80)
I do or do not use risk calculators or nomograms to inform my treatment decision‐making (Memory, Attention and Decision Processes)	Do use: 3 (25) Do not: 2 (17)	Do use: 2 (20) Do not: 1 (10)
**Differences**		
The role of guidelines		
Evidence base, that underpins guidance, does not support ADT before Surgery (Knowledge)	11 (92)	7 (70)
Guidance is evidence based (Knowledge)	9 (75)	6 (60)
Practicing in an academic setting facilitates guideline adherence (Environmental Context and Resources)	0 (0)	6 (60)
The cancer care setting and patients		
I consider multidisciplinary meetings an essential part of cancer care to ratify decision‐making (Behaviour Regulation)	7 (58)	9 (90)
The urologist's beliefs and experience		
ADT has serious side effects that affect the patient (Belief About Consequences)	12 (100)	‐ (‐)
I will offer recommendations for treatment based on the patient's cancer characteristics, health and fitness (Memory, Attention and Decision Processes)	11 (92)	6 (60)
Giving ADT before surgery has (negative or no) implications for the surgery (Belief About Consequences)	Negative: 4 (33) No: 6 (50)	‐ (‐)
My practice around hormones before surgery is in line with my colleagues in the department (Social Influences)	6 (50)	9 (90)
Hormones (ADT) may affect options for other or future treatments (Belief About Consequences)	5 (42)	‐ (‐)

## 4 DISCUSSION

A return to and continuation of established evidence‐based ADT practices, following COVID‐19, is required to ensure patients across Europe receive optimal cancer care.^3^ By applying a behavioural theory‐based approach, key influences on pre‐surgical ADT decision‐making in the UK and Italy, during and following COVID‐19 restrictions, were identified. The identified influences on ADT prescribing were related to the theoretical domains of Behavioural Regulation, Environmental Context and Resources, Goals, Intention, Knowledge, Memory, Attention and Decision Processes, Social Influences and Social/Professional Role and Identity. Belief About Consequences and Emotional influences were identified in the UK‐based participants only. Appropriate behaviour change techniques (BCT) that will form strategies to eliminate inappropriate use of ADT can be determined from these findings.^16^ Further, the assessment of the similarities and differences between countries indicate where strategies could work internationally or where tailoring may be appropriate. Consistent with the IMAGINE pan‐European audit that took place before the pandemic,^6^ generally, participants in both countries reported that they usually do not prescribe neoadjuvant ADT before surgery in their usual practice. However, COVID‐19 presented an exceptional resource‐limited circumstance that, in some cases, prompted a change to ADT practice. This provided an opportunity to understand why non‐guideline adherent ADT practices may escalate in future resource‐limited circumstances and help establish evidence‐based strategies to mitigate this.

Guidelines were found to facilitate appropriate practice as participants, in both countries, accepted the guidance and used it to inform treatment plans, during and beyond COVID‐19. Ensuring an awareness and dissemination of (inter)national guidance, particularly should there be a change, is key to appropriate and safe practice. However, our findings highlight that the legitimacy of the underpinning evidence is a potential for deviation from guidelines. As in both the UK and Italy, some participants highlighted insufficiencies in the current research regarding neoadjuvant ADT. Additionally, participants in both countries reported an authority to depart from guidelines. Healthcare professionals' attitudes towards evidence are a key contributor to the use of unnecessary practices.^24^, ^25^, ^26^ As the volume of patients and pressures on surgical capacity are expected to increase across Europe,^24^ deviations from usual management plans and guidance, as seen during COVID‐19, may occur. Therefore, strategies that provide clear information of the legitimacy of the research and the approaches to take if a deviation is considered could, for example, contain BCT such as information about the health consequences (BCT 5.1), the salience of the consequences (BCT 5.2), to reiterate the evidence and social support (BCT 3.1) to capitalise on the expertise of colleagues regarding a deviation.

Despite similarities of the influence of guidelines in the UK and Italy, the beliefs about the consequences of ADT use diverged. Due to the lack of reference to the consequences of ADT use (during and following COVID‐19), this domain was classified as irrelevant to Italian‐based ADT practice. This lack of influence (or lack of identification of this influence) could be attributed to the fact that the sample of Italian‐based participants had less time in practice, on average (~4 years), and consisted of more registrars (or equivalents) who likely have less experience of the consequences of neoadjuvant ADT use. During COVID‐19, the majority of Italian‐based participants reported that the status quo (i.e., appropriately not using neoadjuvant ADT) remained. This could also be the reason for the lack of relevance found for the Belief About Consequences and Emotion domain in the Italian‐based participants. UK‐based urologists tended to reference severe side effects, with a few mentioning negative implications for the upcoming surgery as reasons not to offer neoadjuvant ADT. This shifted in COVID‐19, as the pathological advantages provided by ADT or the worry of not being able to offer timely treatment justified the use of neoadjuvant ADT. Although the Belief About Consequences domain failed to meet the relevant criteria for the Italian‐based sample in this study, we suggest that strategies targeting the Belief About Consequences should not be exclusive to the UK. Barriers to appropriate practice such as secondary benefits of ADT and facilitators such as ADT causing side‐effects and potentially negatively effecting future treatment are indicated in the literature to be strong sources of influence on ADT use.^21^ ^,^ ^27^ Strategies should contain information about the health consequences (BCT 5.1) to ensure the consequences of ADT are widely known and are not outweighed in resource‐limited circumstances. Equally, should such circumstances arise, having a pre‐established alternative (behavioural substitution, BCT 8.2) such as further counselling or a period of decision‐making time, could ensure ADT is not prescribed unnecessarily.

The setting that urologists work in were influential to ADT prescribing on multiple levels. Italian‐based urologists suggested that academic hospitals were more likely to be guideline adherent, which was attributed to more exposure to research or staff members associated with urological societies/guideline panels. Previously, the type of hospital has been found not to impact appropriate ADT prescribing across Europe^7^; however, the location of the hospital was predictive of ADT non‐adherence in Italy, where patients in central or south Italy were more likely to receive ADT unnecessarily.^8^ Exposure to other staff members (that may have society or guideline association) and their prescribing behaviours is facilitated by multidisciplinary team (MDT) meetings. MDTs were established in both countries, although to varying degrees. MDTs have previously been associated with less use of unnecessary practices, compared to unilateral decision‐making.^25^ Our findings suggest that appropriate ADT practice can be facilitated by team discussions and an awareness of how colleagues practice. This is consistent with previous literature that report decisional support, collaborative working and collegiality can be derived from effective MDTs.^26^ Strategies should capitalise on the social influencing factors that facilitate optimal decision‐making in such a setting, such as Social support (BCT 3.1 and 3.2) and information of other's approval (BCT 6.3).

A shared decision‐making approach was used in both countries and allowed patients to consider and choose their treatment. Patient preferences were heavily influential in a urologist's decision to recommend or prescribe ADT. Our findings purport that patient preferences were driven by anxiety around their cancer diagnosis and timely treatment, which may have influenced neoadjuvant ADT use. This is consistent with the TDF behavioural examination of ADT practices in the United States. Skolarus et al. reported that although inappropriate practice was not common, some urologists were willing to consider using ADT inappropriately to comply with patient's wishes, even when the urologist knew there would be no clinical benefit.^21^ Additionally, in this particular context, a patient would be allowed to switch to the surgery route having previously undergone neoadjuvant ADT in preparation for radiotherapy. Previous literature has suggested that although these treatment changes are a patient's prerogative, more could be done to understand the sufficiency of the patient–healthcare professional interactions in an effort to prevent switching treatment route where ADT has already been offered.^7^ Patients have a large role in the reduction of unnecessary health care practices,^25^ their anxiety, lack of trust or unrealistic view of a practice can prompt the use of care incongruous to guidelines and lead to additional consequences.^28^ Patients should remain a key consideration in strategy development.

### 4.1 Strengths and limitations

This study employed a behavioural science theoretical lens to understand the influences on inappropriate ADT prescribing. The use of a behavioural approach allowed a clear conceptualisation of ADT practice in the UK and Italy, as well as contextualisation within broader ADT overuse literature. Identified barriers and facilitators provide tangible information that can be utilised to develop suitable strategies to address inappropriate ADT practice. Known restrictions of the TDF were mitigated by the inductive coding approach.

This study also had limitations. The interviews were conducted in 2022, when healthcare professionals were still pressured by COVID‐19. This meant that responses to study invitations may have been limited. This may have contributed to lack of availability of European urologists at the time of piloting also, as we were unable to pilot interview with an Italian‐based urologist. Additionally, to accommodate the time limitations urologists were experiencing, we opted for a pragmatic approach in our interview (as opposed to a traditional TDF interview) to ensure that healthcare professionals could participate, in the most time‐efficient manner possible. We covered all TDF domains by asking more ‘general’ questions followed by further questions or prompts to understand beliefs. We appreciate this is different to the traditional TDF‐prescribed structured guide used in previous literature, but believe a semi‐structured interview with questions informed by the TDF in this format lends itself to understanding prominent beliefs in a concise and natural‐conversational way. A further potential limitation was that the interviews were conducted in English. Despite all the Italian‐based participants being bilingual, some phrases or words may not have been understood as intended. Although this was not deemed a significant issue during study, there could have been misconceptions during the interview and the analysis of the Italian data. Finally, in the analysis we did not identify a standard method to establish similarities and differences of TDF beliefs between two countries. The criteria of these features were agreed upon within the research team, based on the sample size and educated opinions of a threshold that may indicate a similarity or difference.

### 4.2 Implications

Guidance, clinical environment, patients and individual urologist's beliefs and experiences that influence ADT prescribing can be used to develop suitable strategies to further reduce or eliminate ADT before surgery in the UK and Italy. COVID‐19 offered an opportunity to understand the changes to these influences in resource‐limited settings, which may be experienced in future as the volume of cancer patients increases. This study highlights similarities and differences between the UK and Italy which offer design and tailoring options for strategies to meet the needs of varying contexts.

## 5 CONCLUSION

ADT prescribing behaviour is influenced by environmental, social and intrinsic factors that should be considered in strategy development to reduce unnecessary ADT use. Theory‐informed strategies to reduce or eliminate inappropriate ADT use will ensure optimal care for patients and efficient use of cancer care resources across Europe.

## AUTHOR CONTRIBUTIONS

Jennifer Dunsmore, Eilidh Duncan, Sara J. MacLennan, James N'Dow, Ted A. Skolarus and Steven MacLennan contributed to study rationale and design. Jennifer Dunsmore, Eilidh Duncan, Sara J. MacLennan, Steven MacLennan and Ted A. Skolarus contributed to materials design. Jennifer Dunsmore, Eilidh Duncan and Steven MacLennan coded or double‐checked coding. Philip Cornford, Francesco Esperto, Nicola Pavan, María J. Ribal and Monique J. Roobol were consulted to sense‐check the results. Jennifer Dunsmore drafted the manuscript. Sara J. MacLennan, James N'Dow, Philip Cornford, Francesco Esperto, Nicola Pavan, María J. Ribal, Monique J. Roobol, Ted A. Skolarus and Steven MacLennan offered comments and feedback on the manuscript.

## CONFLICT OF INTEREST STATEMENT

Monique J. Robool, Steven MacLennan and Jennifer Dunsmore have received an EAU Research Foundation seeding grant. Steven MacLennan and Jennifer Dunsmore secured the University of Aberdeen Elphinstone PhD Student Scholarship and stipend funds from Cancer Research Aberdeen and North East Scotland (CRANES), Registered charity number: SC034542. Philip Cornford is the Chair of the EAU Prostate Cancer Panel and receives consulting fees from AstraZeneca, Ipsen, Janssen; payments or honoraria for lectures/ events from Accord, Bayer and Ipsen; and support to attend meetings from Ipsen, Jassen and Bayer. Francesco Esperto is a board member for YAU endourology and stones group, YOU, SIU and EBU. Nicola Pavan receives consulting fees for Ipsen and Ferring and payments or honoraria for lectures/events from Accord. Ted A. Skolarus received grant from the US National Institutes of Health National Cancer Institute R37 CA222885: De‐implementation of low value castration for men with prostate cancer and participates on the Data Safety and Monitoring Board: US NHBLI U01HL159880 Eliminating Monitor Overuse (EMO) Hybrid Effectiveness‐Deimplementation Trial, Children's Hospital of Philadelphia, Perelman School of Medicine, University of Pennsylvania. James N'Dow, María J. Ribal and Sara J. MacLennan do not have any conflict of interests to declare.

## Supporting information


**Data S1.** Supporting Information.
