# Supplementary material for: Influences on androgen deprivation therapy prescribing before surgery in high‐risk prostate cancer
Source: BJUI Compass. 2024 Jul 14;5(9):874–84. doi: 10.1002/bco2.411 (PMC11420097; doi:10.1002/bco2.411)
Supplement: Supplementary file 1 — Data S1. Supporting Information. [file BCO2-5-874-s001.docx]

Interview schedule

Introduction:

Thank you for agreeing to participate in this interview. This should take about 45 minutes, however if you need to stop at any time please let me know.

This is for my PhD project, I am interested in learning more about the treatment of high risk localised prostate cancer patients over the pandemic. I’m not here to evaluate clinical practice, I would just like to learn more about practice in across Europe. I want to let you know I’m not medically trained, so it would be great if you could talk to me in lay terms please.

I have an interview schedule to help me to remember to cover the relevant points. What you tell me will remain confidential, your transcript, as well as any quotations if used in any report or presentation, will be anonymous. This research has been approved by the University of Aberdeen College Ethics review board.

Do you have any questions before starting?

[Start recorder]

Interview content:

OK, to start, just a couple quick questions:

1. What is your current job title?
2. How long have you been in this role?
3. What country do you work in?
4. [Consultant] How many non-metastatic prostate cancer patients do you currently under your care?
5. [Registrar] How many patients currently in your department have non metastatic prostate cancer? (Prompt: high/low volume)

I am aware that the COVID-19 pandemic changed the way healthcare was delivered.

I would like to understand *if* and *how* COVID changed practice and treating prostate cancer and if those changes continue or impact how these patients are treated today. I refer to the ‘pandemic’, please think of the year of 2020, however I know things may have changed multiple times within 2020 and beyond so feel free to mention anything you think is relevant at any time during the last 2 years of this pandemic.

I would like to ask you about your treatments for patients diagnosed with *high risk localised prostate cancer,* defined as those with*:* PSA over 20, GS over 7, stage T2c.

1. How would you treat a high risk localised prostate cancer patient today? (nb. usually RP, Radio) [Memory, attention and decision processes]
2. And how would you have treated this patient during 2020 of the pandemic? [Memory, attention and decision processes]
3. [Does this differ from how you would treat this patient today?] [Intention]
4. [if different] Why was the treatment different back then? [Belief about consequences, Environment]
5. [does not mentioned ADT] Would you have considered ADT for this patient? Why/ why not? [Intention]
6. What would happen to this patient if you did/ did not give ADT? [Beliefs about consequences, Goals]
7. [To all] Thinking about what may have influenced your treatment decision making, was there anything COVID brought about that affected this? [All domains]
8. What were the additional considerations you had to make? [Memory, attention and decision processes]
9. Were there any changes were there to the *way this patient was diagnosed,* during COVID that may have influenced your decision? e.g. MDT, waitlists [social influences, environmental context and resources, Memory, attention and decision processes]
   1. Were these patient prioritised differently during COVID? [Goals]
   2. Are these changes still in place today? Do they affect you?
   3. [if answer ‘yes’] How do these changes impact your treatment decisions today? [Goals]
10. Would this decision usually be discussed with your colleagues? (n.b: MDT [social influences, Reinforcement]
    - 1. Did this process change due to the COVID situation?
      2. What is this process like today?
      3. Does this impact your treatment decisions today? [Social influences, Reinforcement]
11. Did you find these treatment decisions difficult to make in 2020? [Skills, Belief about capabilities]
    1. How so?
    2. Was it stressful? [Emotion]
12. What about patient’s views and expectations in this time? [Social influences]
    1. How involved would the patient have been in this decision in 2020?
    2. Is the patients involvement different today?
13. *(If not mentioned guidance before this)*: Was there anything else you consulted to make this decision? [All domains]
14. *If guidance mentioned*: What guidance did you refer to make this decision? [Knowledge]
    1. Was this guidance different from guidance available pre-COVID? [Knowledge]
    2. Are you aware of any COVID specific guidance? [Knowledge]
    3. How did the guidance differ? [Knowledge]

(prompt) in relation to treatment option? [Knowledge]

- 1. How acceptable did you find this ‘COVID’ guidance? [Social professional role and identity]
  2. Is this guidance still applicable for today?

1. What changes made in COVID ‘times’ continue or still impact today? [All domains]
   1. Has your practice ‘reverted’ back to pre-pandemic standard (or ways)? [Behavioural regulation]

Now I just have 6 final questions regarding the use of Androgen Deprivation Therapy to finish up.

1. Do you think *your* use of ADT has changed because of COVID? [Social professional role and identity, Intention]
   1. How about use in *your department*? [Social influences]
2. [may have covered] Can you tell me of any scenarios why deciding to use ADT in high-risk localised patient may have been acceptable in the initial COVID wave? [Social professional role and identity, Memory, attention and decision processes, Intention]
3. Thinking of today, can you tell me of any scenario where prescribing ADT to high risk localised prostate cancer patient before surgery today is acceptable? [Social professional role and identity, Intention]
   1. Why do you think that may happen?
   2. What about the thoughts of Urologist who may do this?
   3. How about in the ‘world’ of Urology?
4. What alternatives to ADT could be considered at this time? [Optimism]
5. Can you tell me of a scenario where prescribing ADT before surgery to a locally advanced prostate cancer (GS Any, PSA any, Stage T3 to 4 or load positive) patient may be acceptable? [Social professional role and identity, Memory, attention and decision processes, Intention]
6. What would have happened to this patient if they were/ were not given ADT? [belief about consequences, Goals]
7. What are the consequences for you if you prescribe ADT to a locally advanced prostate cancer patient? [Reinforcement]
8. What do you think about the current (i.e. not COVID specific) guidelines around ADT in the non-metastatic PCa setting are…
   1. … acceptable? [Social professional role and identity]
   2. … generally followed in your country? [Social influences]
   3. Why do you think your country follow guidelines? [Behavioural Regulation]
9. Have you noticed any changes in the frequency in the use of ADT before surgery in the time you have practiced? [Reinforcement]
   1. Why do you think that is? [Behavioural Regulation]
   2. What do you think helped solve this issue? [Behavioural Regulation]

Finish of interview:

That’s the end of the interview, is there anything else you would like to add?

[Turn off recorder]

-----END-----

Table S1. TDF domains and definitions (reproduced from Cane et al. 2012 [22])

| TDF Domains | Definitions |
| --- | --- |
| 1. Behavioural Regulation | Anything aimed at managing or changing objectively observed or measured actions |
| 1. Knowledge | An awareness of the existence of something |
| 1. Skills | An ability or proficiency acquired through practice |
| 1. Beliefs about Capabilities | Acceptance of the truth, reality, or validity about an ability, talent, or facility that a person can put to constructive use |
| 1. Beliefs about Consequences | Acceptance of the truth, reality, or validity about outcomes of a behaviour in a given situation |
| 1. Intentions | A conscious decision to perform a behaviour or a resolve to act in a certain way |
| 1. Reinforcement | Increasing the probability of a response by arranging a dependent relationship, or contingency, between the response and a given stimulus |
| 1. Goals | Mental representations of outcomes or end states that an individual wants to achieve |
| 1. Memory, Attention and Decision-Processes | The ability to retain information, focus selectively on aspects of the environment and choose between two or more alternatives |
| 1. Environmental Context and Resources | Any circumstance of a person's situation or environment that discourages or encourages the development of skills and abilities, independence, social competence, and adaptive behaviour |
| 1. Social Influences | Those interpersonal processes that can cause individuals to change their thoughts, feelings, or behaviours |
| 1. Optimism | The confidence that things will happen for the best or that desired goals will be attained |
| 1. Emotions | A complex reaction pattern, involving experiential, behavioural, and physiological elements, by which the individual attempts to deal with a personally significant matter or event |
| 1. Social/Professional Role and Identity | A coherent set of behaviours and displayed personal qualities of an individual in a social or work setting |

| **Table S2. Main similarities and differences of beliefs across the UK and Italy for COVID-19 practice** | | | | | |
| --- | --- | --- | --- | --- | --- |
| **Similarities** | | | | | |
| **The role of Guidelines and evidence** | | **UK n(%)** | **Italy n(%)** | **Illustrative Quotations from Urologists based in the UK** | **Illustrative Quotations from Urologists based in Italy** |
|  | COVID-19; I was or was not aware of a change to treatment management guidance (Knowledge) | 11 (92) | 10 (100) | Well, that was the advice given to us by BAUS that in high-risk patients where there’s going to be a substantial delay then they had suggested that [neoadjuvant ADT] would be their guidance, in a way, to manage that delay. (Interview 11, UK) | I’m not aware of changes really for the pandemic. (Interview 13, Italy) ---contrasting view---I think that there were some recommendation but not definitive mandation, because the situation of the hospital was so jeopardised that I think did not definite suggestion that be possible (Interview 22, Italy) |
|  | COVID-19; We will only offer hormones or follow COVID-specific guidance if or when needed (Intention) | 10 (83) | 7 (70) | But I think we resorted to it only when we needed it, but that’s not the mainstay now. (Interview 8, UK) | We consider[ed] the option but we are not using them right now (Interview 22, Italy) |
|  | COVID-19; We prioritised patients according to available Prioritisation Guidance for surgical patients (Environmental Context and Resources) | 9 (75) | 7 (70) | it is from the NHS [redacted] which came in that to every guidance, every organisation saying that, “We are going to have this issue for next three to six month so each team need to identify the cancer which are aggressive enough which is going to kill them or make them more inoperable over the next three months, then they should be the priority”. (Interview 3, UK) | [new guidance/ publications] said in which condition you could delay surgeries and in which not, and who you had to still give the high priority for surgery. (Interview 15, Italy) |
| **The cancer care setting and patients** | | | |  |  |
|  | COVID-19; COVID-19 did or did not affect surgical capacity or resources (Environmental Context and Resources) | 10 (83) | 10 (100) | We also didn’t have the capacity because we went from having 25 lists a week to having 3 lists a week, so we didn’t have the capacity to operate on these patients, so we had to find a way to keep them safe until we could operate on them (Interview 2, UK)  ---other view---  we’re fortunate to be a high-volume centre with two robots.... We picked up the work from the rest of the region as well and the reality is, although it might sound unusual because we were able to continue to operate throughout, so we did... in terms of the Covid pandemic we were able to keep the service running (Interview 4, UK) | Covid didn’t change my management (Interview 13, Italy) --- contrasting view----R- how long was surgery not available for?  P – It was not available at all, only for emergency or urgency, in the first period, for at least six months. (Interview 14, Italy) |
|  | COVID-19; Patients, for the most part, were understanding of the changes made to treatment options (Social influences) | 6 (50) | 5 (50) | It is strange that most patients were quite happy with [the changes] (Interview 9, UK) | patients were well aware that the pandemic was changing their options to a smaller list of options… Everybody was quite understanding, well, the majority of patients were quite understanding (Interview 20, Italy) |
|  | COVID-19; Asides from the pandemic, resources were allocated to cancer care (Environmental Context and Resources) | 5 (42) | 6 (60) | when the pandemic obviously came into picture a lot of the resources were diverted to pandemic but cancer care still was a priority (Interview 6, UK) | we stopped with all the surgery that was not necessary or that can be deferred, but cancer surgeries were still ongoing in our unit since it’s mainly oncology. (Interview 21, Italy) |
|  | COVID-19; Cancer targets relating to treating prostate cancer were or were not 'dropped' during COVID-19 (Behavioural regulation) | 3 (25) | 2 (20) | They were very quickly dropped because they’re a challenge to meet and they’re not always clinically relevant. But now they’re back on, so we have to meet them again. (Interview 5, UK) | We are trying to provide surgery for everyone in less than 50 days, so unless it’s a very serious cancer or something, for prostate cancer, we follow the day of the diagnosis, but this changes during the Covid period. (Interview 14, Italy) |
|  | COVID-19; We used private hospitals to offer surgery to mitigate any delays (Environmental Context and Resources) | 2 (17) | 2 (20) | we got moved to full-time...to the private hospital during the backlog and were there for just over a year doing robotic prostatectomy the whole time. Yes, no real change in what we offered. (Interview 11, UK) | [departments] found ways to reorganise, perhaps asked for some help from the private sector, and it reopened the options for surgical treatment for patients. (Interview 20, Italy) |
| **Differences** | | | | | |
| **The role of Guidelines and evidence** | |  |  |  |  |
|  | COVID-19; Guidelines specified use of Hormones (ADT) before surgery (Knowledge) | 11 (92) | 1 (10) | it was just the sort of guidance about consider putting [patients] on hormones (Interview 2, UK) | the indication in selected patients for neoadjuvant therapy with hormone therapy (Interview 16, Italy) |
|  |  |  |  |  |  |
|  | COVID-19; Guidance was or was not made available to help mitigate any delays due to COVID-19 (Environmental context and resources) | 10 (83) | 6 (60) | BAUS oncology did provide guidelines, so that there was a schedule for teams that were managing prostate cancer and one of the options that was proposed was that you could put patients onto androgen deprivation therapy (Interview 4, UK) | [EAU guidelines] did publish something about prioritising surgery... how to manage emergency situations in patients with positive Covid... for cancer [management] there wasn’t that much change. (Interview 21, Italy) |
|  |  |  |  |  |  |
|  | COVID-19; Anaesthetic Guidelines delayed surgery to ensure 7 weeks of recovery from COVID-19 (Environmental context and resources) | 4 (33) | 0 (0) | I think the current guidelines are to wait seven weeks from a Covid infection to then having anaesthetic treatment. That does introduce delays. Because it’s seven weeks, and we know it’s a finite time, we’ve not given patients additional treatment (Interview 5) | - |
|  |  |  |  |  |  |
| **The cancer care setting and patients** | | | |  |  |
|  | COVID-19; Eligible patients were not given equal option between Surgery or Radiotherapy treatments (Environmental context and resources) | 9 (75) | 4 (40) | We were offering then treatment was low, diagnostic was low, the treatment options were different and again, not equitable as we did before or now (Interview 3, UK)  ---other view---  Broadly speaking we offer treatment options with equipoise to our patients, so they have a choice between radiotherapy or surgery and all things being balanced.  (Interview 12, UK) | I would say that we were forced to use [hormones] more by the fact that we were missing the complete array of treatments for prostate cancer. (Interview 20, Italy) |
|  |  |  |  |  |  |
| **The urologist's beliefs and experience** | | | |  |  |
|  | COVID-19; I did or did not consider Hormones (ADT) where delays in surgery were experienced or anticipated (Environmental context and resources) | 10 (83) | 4 (40) | we’re giving it to our patients because of the delay. (Interview 11, UK) | patient who cannot have access to the operating room... the only thing I can do is give him hormone therapy (Interview 19, Italy) |
|  |  |  |  |  |  |
|  | COVID-19; Other cancers or illnesses were prioritised over Localised Prostate can (Goals) | 9 (75) | 5 (50) | So prioritisation in terms of disease obviously came into picture, so prostate cancer by definition was probably the least of priority amongst the cancers. (Interview 6, UK) | The bladder cancer and the renal cancer were treated before high-risk prostate cancer (Interview 18, Italy) |
|  |  |  |  |  |  |
|  | COVID-19; I think that other Urologists in my country practiced the way I did in COVID-19 (Social Influences) | 9 (75) | 3 (30) | R- do you think this is same image across the UK? P - As far as I can understand, yeah. (Interview 3, UK) | I know that other centres did it, but for us, we didn’t do this. (Interview 14, Italy) |
|  |  |  |  |  |  |
|  | COVID-19; ADT could prevent the cancer from getting worse while surgery was delayed (Belief about consequences) | 8      (67) | -       (-) | Doing nothing is not acceptable and waiting for a chance to takeover cancer is not a good way of practicing. Meanwhile, because we know that hormones can control the disease to a certain extent. (Interview 9, UK) | - |
|  |  |  |  |  |  |
|  | Giving ADT before surgery allows you to buy time (Belief about consequences) | 5 (42) | - (-) | you can buy time with these patients (Interview 9, UK) | - |
|  |  |  |  |  |  |
|  | COVID-19; There remained an urgency to treat High risk prostate cancer (Goals) | 6 (50) | 8 (80) | for high-risk patients you want to get on and do [surgery] as quickly as possible, and so the fear was that we didn’t know when we would be able to start surgery. (Interview 5, UK) | all the focus was on the patient with higher risk disease (Interview 17, Italy) |
|  |  |  |  |  |  |
|  | COVID-19; I found treatment decisions during COVID-19 stressful (Emotion) | 4 (33) | - (-) | R - Did you find these treatment decisions difficult to make during the pandemic?  P - Yes, yeah, because I might say that, “Oh, he’s an inpatient and he should have surgery”, but then because there were no resources so we had to change that (Interview 3, UK) | - |
|  | COVID-19; I felt it was risky to make patients wait without treatment (Emotion) | 3 (25) | - (-) | A bit like the reason I started two patients on it because I was worried they wouldn’t get their surgery in fact.  (Interview 1, UK) | - |
|  | COVID-19; I worry that offering hormones (ADT) caused harm (Emotion) | 3 (25) | - (-) | I think there’s an element of guilt, there’s an element of fear you know, what if we get it wrong... to some extent playing god and you don’t feel that you’re the person to make that decision. There are some moral challenges that you go through, you question yourself a fair bit... there is of course challenges there. (Interview 6, UK) | - |

| **Table S3. Main similarities and differences of beliefs across the UK and Italy for usual practice** | | | | | |
| --- | --- | --- | --- | --- | --- |
| **Similarities** | | | | | |
| **The role of Guidelines and evidence** | | **UK n(%)** | **Italy n(%)** | **Illustrative Quotations from Urologists based in the UK** | **Illustrative Quotations from Urologists based in Italy** |
|  | I believe the guidelines are followed in my country (Social influence) | 12 (100) | 10 (100) | R- Do you think that these guidelines are generally followed in the UK? P - I think on the whole, yeah. I think most urologists use that as a template for their clinical practice (interview 12, UK) | R- Do you think they’re generally followed in your country? P - It depends... were not so homogenous in the country (Interview 16, Italy)---contratsing view---R - And do you think these guidelines are generally followed in your country? P – Yes. (Interview 21, Italy) |
|  |  |  |  |  |  |
|  | My ADT practice is informed by guidelines (Behavioural regulation) | 12 (100) | 10 (100) | NICE guidance, the EAU guidance, are probably the two things that we use (interview 4, UK) | the EAU guidelines are very, very useful for my decision-making and the treatment of prostate cancer (Interview 18, Italy) |
|  | There are no alternatives to ADT before surgery (Knowledge) | 12 (100) | 7 (70) | R- Is there any alternatives to hormone therapy that could be offered before surgery? P – I think nothing with a strong evidence base that shows improved outcomes.  (Interview 5, UK) | Are there any alternatives that could be offered before surgery instead of hormones? P – No. (Interview 21, Italy) |
|  |  |  |  |  |  |
|  | I find usual guidance is acceptable (Social/ Professional role and Identity) | 10 (83) | 10 (100) | I’m a big believer in using the EAU guidelines because I know that they’re up to date, robust, look at all the evidence very thoroughly, so I’m very happy to use them. (Interview 10, UK) | I think [the guidelines] are acceptable, yes. I trust EAU guidelines of course. (Interview 22, Italy) |
|  |  |  |  |  |  |
|  | I am allowed to deviate from the guidance if required (Social/ Professional role and Identity) | 6 (50) | 5 (50) | A guideline is there to explain to a clinician what may be an acceptable course of practice: it is not necessarily what should be applied to all patients. It’s a guidance, it’s not mandated. (Interview 2, UK) | I would say real life clinical scenarios do not always adhere to guidelines, so you always have to keep in mind other things regarding the patients… then you have to adapt the guidelines (Interview 20, Italy) |
|  | Updated research should be conducted in the Neoadjuvant setting (Knowledge) | 4 (33) | 3 (30) | the role of neoadjuvant hormones prior to surgery, which did show that it did not improve overall survival in quotes, because they did not follow these patients long enough, so we never knew about it. The trial wasn’t powered to see that but what it definitely showed that it reduced the risk of positive margins. But the issue with positive margins is they do not necessarily translate into disease progression and that’s based on more recent evidence (Interview 9, UK) ----other view----we’re into this space where there’s very little evidence and I personally wouldn’t offer anything else and none of the guidelines that are applicable to the UK practice advocate anything else (interview 12, UK) | Actually, there’s not much evidence. It is not that it doesn’t work, but there’s not so much evidence to do it. (interview 19, Italy) ---other view--- all the studies regarding hormonal therapy before surgeries were with older therapies ...I guess that it could be valid treatment option in a neoadjuvant setting between surgery, but the only thing that we can do is to investigate this type of treatment in a strictly structured protocol. (Interview 17, Italy) |
|  |  |  |  |  |  |
| **The cancer care setting and patients** | | | |  |  |
|  | Patients have the ultimate decision/ heavily influence on their treatment (Social Influence) | 11 (92) | 9 (90) | The decision is made ultimately by the patient, but we make recommendations according to what we’re able to offer or what we think is appropriate to offer. (Interview 5, UK) | At the end, the decision has to be [from] them, ... what I usually suggest depends on the expectation of the patients. (Interview 15, Italy) ---other view--- the patients will choose which one is the best for him. (Interview 19, Italy) |
|  |  |  |  |  |  |
|  | Treatment options for every cancer patient are discussed in an MDT meeting (Environmental context and resources) | 10 (83) | 10 (100) | we have a multi-disciplinary team meeting once a week and every cancer patient is discussed. (interview 2, UK) | We have a multi-disciplinary team and not all patients, but, yes, we particularly discuss the majority of cases. (Interview 13, Italy) |
|  | ADT before surgery could be considered in a clinical trial (Environmental context and resources) | 4 (33) | 4 (40) | In the PROTEUS study men are randomised to androgen deprivation therapy and placebo versus androgen deprivation therapy and apalutamide. If they’re in that study that’s the only time that we would have given androgen deprivation therapy.  (Interview 4, UK) | we are a recruiting group for clinical trial, PROTEUS, that is the only reason we give hormone therapy before surgery. (Interview 15, Italy) |
|  |  |  |  |  |  |
| **The urologist's beliefs and experience** | | | |  |  |
|  | I do not offer ADT before surgery in my usual practice (Intention) | 10 (83) | 7 (70) | the use of hormones before surgery was not an option before the pandemic (Interview 9, UK) | I never will use hormone therapy before the surgery (Interview 16, Italy) |
|  | Other urologists may or may not have justifiable reasons to use ADT before surgery (Social Influences) | 9 (75) | 9 (90) | [urologists prescribing unnecessary ADT] need to review their practice and change it (Interview 1, UK)----Contrasting view----I think with the right indication, with a strong indication, then it’s not unreasonable…. take into account your local patient cohort (Interview 10, UK) | there are still some urologists that think that using hormones cause a regression, a local regression of the pathology and, so the surgery became easier okay (Interview 16, Italy) ---other view--- the old surgeons still think that it could work for high-risk patients (Interview 19, Italy) |
|  |  |  |  |  |  |
|  | ADT before surgery has not be a routine treatment in the time or places I have practiced (Environmental context and resources) | 9 (75) | 8 (80) | I’ve been in Urology for twenty years... now. No, ADT before surgery routinely for localised high-risk prostate cancer is not something me or any of the colleagues I work with have done routinely. (interview 7, UK)   ---Contrasting view--- I started training not that long ago, and even then most patients who had prostate cancer would be on hormones, whether that’s people who had surgery or... radiotherapy… hormones were very, very common…but now the proportion of people who are on hormones is much, much smaller. (Interview 10, UK) ---Contrasting view---we had a six-month waiting time... we use [neoadjuvant ADT] if there’s going to be a really substantial delay, otherwise we don’t use [neoadjuvant ADT]. (Interview 11, UK) | I haven’t experience for this treatment before surgery (Interview 18, Italy) ---contrasting view---I found during my few years I’ve been practicing, some urologists still prescribing hormones in this scenario, but I would say that it’s not the case generally (Interview 20, Italy) |
|  |  |  |  |  |  |
|  | I do or do not use risk calculators or nomograms to inform my treatment decision making (Memory, attention and decision processes) | 5 (42) | 3 (30) | At the end of the day these models are algorithms, that’s all they are, and modern day algorithms will not incorporate everything. [treatment recommendations] don’t go down to any validated or prescribed model. (Interview 6) ----Opposing view---- Predict Prostate... gives out a percentage of difference and preference whether surgery versus... not surgery...That is what we use to build our decision on (Interview 10, UK) | We use the Memorial Sloan Kettering cancer nomograms to define the risk, and on this nomogram, we decide when and the time to treat these patients. (Interview 18, Italy) ---contrasting view--- R- Do you refer to any tools or information when prioritising or stratifying these patients?  P –No, we don’t. (Interview 19, Italy) |
|  |  |  |  |  |  |
| Differences | | | | | |
| **The role of Guidelines** | |  |  |  |  |
|  | Evidence base, that underpins Guidance, does not support ADT before Surgery (Knowledge) | 11(92) | 7 (70) | There’s no evidence that it improves the outcomes in surgery, which is why we don’t give it (Interview 1, UK) | [neoadjuvant use of ADT before surgery] was not approved by the guidelines, it was not supported by the evidence from the literature (Interview 14, Italy) |
|  | Guidance is evidence based (Knowledge) | 9 (75) | 6 (60) | I mean if the guidance is robust, and the process for developing the guidance is robust, and it’s based on the best available evidence with valid interpretation, that’s very useful (interview 5, UK) | You can follow [guidelines] because you have a lot of evidence...summarised and you have recommendations which are easy to follow. (Interview 13, Italy) |
|  |  |  |  |  |  |
|  | Practicing in an academic setting facilitates guideline adherence (Environmental context and resources) | 0 (0) | 6 (60) | N/A | it depends if you’re working at a university hospital or in a non-university hospital. So, I’ve always worked in a university hospital, so I’ve always followed guidelines because this is the way I was [trained] (interview 15, Italy) ---- other view --- [academic centres] are involved in all the research process and on studies which this recommendation are based, and also ... if you have a higher surgical volume, if you have more experience in dealing with high risk patient... it is easier to follow the guidelines (interview 17, Italy) |
|  |  |  |  |  |  |
| **The cancer care setting and patients** | | | |  |  |
|  | I consider multidisciplinary meetings an essential part of cancer care to ratify decision making (Behaviour Regulation) | 7 (58) | 9 (90) | It’s essential. No patient goes through the prostate cancer pathway without an MDT discussion. (Interview 8, UK) | How impactful do you find these MDT discussions on your treatment decisions? P - For selected patients it’s fundamental (Interview 16, Italy) -----other view----- We moved to an MDT-centred approach... four to five years ago...now all these cases are assessed by the MDT, and that is the cornerstone of our practice when it comes to offering the best possible treatment (Interview 20, Italy) |
|  |  |  |  |  |  |
| **The urologist's beliefs and experience** | | | |  |  |
|  | ADT has serious side effects that affect the patient (Belief about Consequences) | 12 (100) | - (-) | we’re tampering with the patient’s hormones, the imbalances that causes leads to lots of side effects, general side effects in the form of hot flushes, sweats, fatigue, nausea and vomiting, feeling generally unwell. Those are all side effects of hormones. Yes, mainly the hot flushes and some men develop gynecomastia, which is feminisation of their breasts and yes, those are some side effects of hormones. (Interview 8, UK) | - |
|  |  |  |  |  |  |
|  | I will offer recommendations for treatment based on the patient's cancer characteristics, health and fitness (Memory, Attention and Decision processes) | 11 (92) | 6 (60) | [the treatment recommendation] really does depend on the fitness of the patients, and their capabilities. (Interview 4, UK) | [treatment options] usually depends on the age and how fit the patient is (Interview 14, Italy) |
|  |  |  |  |  |  |
|  | Giving ADT before surgery makes or does not make the surgery more challenging (Belief about Consequences) | 10 (83) | - (-) | I don’t think there’s any consequences from the surgical point of views, just consequences to the patient. (interview 1, UK)  ---other view----  starting hormones caused an immune reaction and that made the surgery slightly difficult.  (interview 3, UK) | - |
|  |  |  |  |  |  |
|  | My practice around Hormones before surgery is in line with my colleagues in the department (Social Influences) | 6 (50) | 9 (90) | ADT before surgery routinely for localised high-risk prostate cancer is not something me or any of the colleagues I work with have done routinely (interview 7, UK) | R – would you say [your practice is] the same for the urologists in your department? P – Yes (Interview 20, Italy) |
|  |  |  |  |  |  |
|  | Hormones (ADT) may affect options for other or future treatments (Belief about consequences) | 5 (42) | - (-) | Biologically at some point they can become refractory [does not respond to treatment] and patients will stop responding to them. So the lesser an individual is exposed to it in the event that they really need it in the future the better, is the view I take. (Interview 6)---contrasting view---  Starting with hormones… doesn’t preclude too many treatment options (Interview 12) | - |
